# Metabolomics Meets Nutritional Epidemiology: Harnessing the Potential in Metabolomics Data

**DOI:** 10.3390/metabo11100709

**Published:** 2021-10-19

**Authors:** Lorraine Brennan, Frank B. Hu, Qi Sun

**Affiliations:** 1 Conway Institute, Institute of Food and Health, School of Agriculture and Food Science, UCD, Belfield, Dublin 4, Ireland; 2 Channing Division of Network Medicine, Department of Medicine, Brigham and Women’s Hospital and Harvard Medical School, Boston, MA 02115, USA; nhbfh@channing.harvard.edu (F.B.H.); qisun@hsph.harvard.edu (Q.S.); 3 Department of Nutrition, Harvard TH Chan School of Public Health, Boston, MA 02115, USA; 4 Department of Epidemiology, Harvard TH Chan School of Public Health, Boston, MA 02115, USA

**Keywords:** metabolomics, dietary intake, biomarkers, dietary patterns, metabolites

## Abstract

Traditionally, nutritional epidemiology is the study of the relationship between diet and health and disease in humans at the population level. Commonly, the exposure of interest is food intake. In recent years, nutritional epidemiology has moved from a “black box” approach to a systems approach where genomics, metabolomics and proteomics are providing novel insights into the interplay between diet and health. In this context, metabolomics is emerging as a key tool in nutritional epidemiology. The present review explores the use of metabolomics in nutritional epidemiology. In particular, it examines the role that food-intake biomarkers play in addressing the limitations of self-reported dietary intake data and the potential of using metabolite measurements in assessing the impact of diet on metabolic pathways and physiological processes. However, for full realisation of the potential of metabolomics in nutritional epidemiology, key challenges such as robust biomarker validation and novel methods for new metabolite identification need to be addressed. The synergy between traditional epidemiologic approaches and metabolomics will facilitate the translation of nutritional epidemiologic evidence to effective precision nutrition.

## 1. Introduction

Traditionally, nutritional epidemiology is the study of the relationship between diet and health and disease in humans at the population level. In this case, food intake is the exposure of interest, and it has been studied in relation to a wide range of health and disease outcomes. Classically, epidemiologists examine how dietary intake impacts the occurrence of disease through collection of data in a large population and comparing groups within this population for disease incidence. Statistical approaches are used to estimate the extent to which the exposure influences the risk of disease in the population. The measures are usually associations, and causal or mechanistic underpinnings are difficult if not impossible to establish in such approaches. Notwithstanding this, nutritional epidemiology studies play an important role in the development of policies on diet, health and disease and in the guidance of fortification policies [1]. With the widespread uptake of omic technologies, nutritional epidemiology has moved from a “black box” approach to a systems approach where genomics, metabolomics and proteomics are providing novel insights into the interplay between diet and health [2,3]. Due to the close interplay between food and metabolism, the application of metabolomics in nutritional epidemiology has been particularly successful. The present review will present key areas where incorporation of metabolomics into nutritional epidemiology has been successful and has helped address some pertinent drawbacks of the traditional approaches.

## 2. Addressing Limitations of Self-Reported Dietary Intake Data

Nutritional epidemiological studies with large numbers of participants have often relied on self-reported instruments to assess dietary intake. Although these instruments such as food frequency questionnaires (FFQs) have been useful to estimate habitual dietary intakes and characterize dietary patterns in free-living populations, it is well documented that these instruments are prone to random and systematic errors. Examples of such errors include underestimated energy intake, recall inaccuracies or biases and difficulty in assessment of portion sizes [4,5,6]. These errors can result in reduced power and underestimated or even inflated associations which in turn partly contributes to the inconsistencies in results in nutritional epidemiology [7,8]. Other instruments such as 24-hr recalls are also subject to recall inaccuracies and random within-person variabilities. The diet record method, which is considered the most accurate self-reported dietary assessment method, is burdensome and expensive and thus often infeasible in large epidemiological studies. To address some of the concerns surrounding self-reported dietary data, the potential role of dietary biomarkers has emerged. While classical biomarkers for salt, protein and energy intake have existed for years, the emergence of metabolomics has resulted in the expansion of dietary biomarkers to include biomarkers for specific foods and dietary patterns [9,10,11]. While the potential of classical biomarkers for correcting self-reported data is well-established, there is emerging evidence that novel food and nutrient biomarkers that are discovered agnostically through metabolomic profiling can also be employed to correct self-reported data.

Lampe and colleagues illustrated the potential of candidate biomarkers for dietary assessment in terms of nutrient intake [12]. Using a series of dietary biomarkers including carotenoids, tocopherols, folate, and vitamin B12, the authors demonstrated that these biomarkers performed as well as the established biomarkers of energy (doubly labelled water) and protein (urinary nitrogen) in estimating nutrient intake. These and other biomarkers have the potential to be used to calibrate self-reported data in large studies [12]. Extension to include the calibrated intakes into regression models allows diet–disease associations to be explored [13].

Our previous work developed calibration equations for citrus intake, derived from feeding trials, using the biomarker proline betaine; the results demonstrated that these equations could be used to calibrate self-reported citrus intake data [14]. As more biomarkers of food intake are validated, the potential for such approaches will open up new avenues for assessing the relationship between food intake and health outcomes.

A recent review of 244 studies identified 69 metabolites that were classed as potentially useful biomarkers of food intake, and these covered fruits, vegetables, meat, legumes, coffee high-fibre foods and seafood [10]. Many of these now need to be validated against the criteria developed by the European consortium focusing on food-intake biomarkers called FoodBall [15]. The criteria include assessment of biological plausibility, time–response, dose–response, robustness, reliability, stability, and analytical performance of the method used to measure them. A series of systematic reviews for a range of foods including meat, green leafy vegetables, cereal foods, apple, pear and stone fruit were recently published [16,17,18]. It should be noted that many of the food-based metabolomics biomarkers are not specific to individual foods or food groups, and thus accurate quantification of intakes of these foods is not possible. At this point, metabolomics is not yet able to accurately distinguish between most specific foods. Therefore, it can be used in conjunction with existing dietary assessment methods to measure dietary intake and assess compliance to dietary interventions, but it is not sufficient to replace the established dietary assessment methods [19].

### Dietary Patterns

There is growing interest in assessment of overall diet, and consequently, biomarkers that reflect dietary patterns. Biomarkers have been successfully used as a surrogate for adherence to predefined specific dietary patterns such as the Mediterranean diet [20,21,22]. Fasting plasma metabolites can distinguish between low and high Mediterranean Diet Score (MDS). Furthermore, serum metabolite levels in postmenopausal women were capable of distinguishing between low and high adherence to four healthy diet scores (the alternate Mediterranean diet score (aMED), alternate Healthy Eating Index (aHEI)-2010, DASH (Dietary Approaches to Stop Hypertension diet, and the Healthy-Eating Index (HEI)-2015) [21].

A study of 2208 men and women identified 65 metabolites that were associated with at least one of the three dietary patterns examined (AHEI, DASH and MDS) [23]. A shared signature of 24 metabolites was associated with all three healthy dietary patterns. Many more examples exist where dietary patterns are correlated to metabolites, and these lay the foundations for the potential of biomarkers to correct self-reported data in order to obtain dietary patterns.

A recent example identified biomarker signatures of dietary patterns, and used the biomarkers for development of calibration equations to address measurement error associated with self-reported data [24]. Although this work included traditional biomarkers, the concepts could be used with metabolomic derived biomarkers. Using a controlled feeding study, associations were found between the biomarker panel and HEI-2010 and aMED. Subsequently calibration equations were created for FFQs, 4-day food diaries and 24 h recall data and could be used for the HEI-2010 dietary pattern. The calibration equations were also developed for the aMED and 4-day food diaries and 24 h recall data. These calibration equations could be used to calibrate intake data and support the study of diet–disease relationships in large cohorts.

Using panels of biomarkers to assign individuals into dietary patterns with no reliance on self-reported data is also attractive. While further work is needed to develop this concept, there are some examples that demonstrate the potential and support additional work in this area. Employing a controlled intervention design, Garcia-Perez and colleagues developed a model based on urinary metabolomics data that could classify individuals into dietary patterns, which was validated in two separate population groups [25]. Using urinary metabolomic data only, individuals were classified into four dietary patterns: replication was achieved in a separate group with good reproducibility over four time points [26]. Further work is needed to expand these approaches and develop them for use in a range of population groups.

## 3. Using Metabolites to Inform about Metabolic Processes

**Figure 1:**
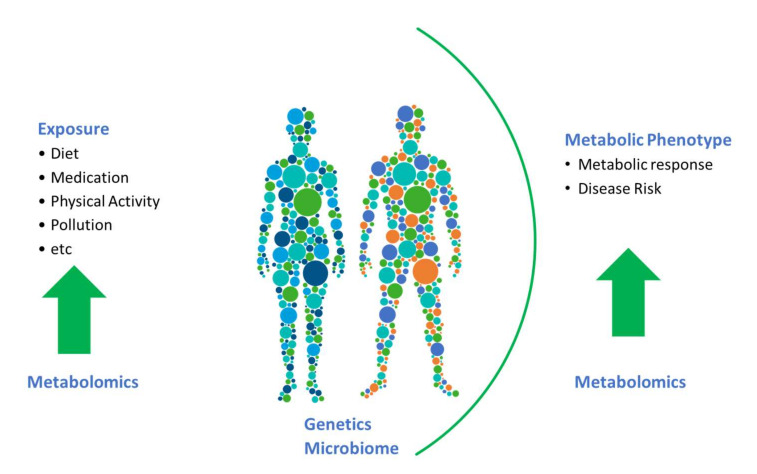
In nutritional epidemiology studies, metabolomics can aid in the assessment of exposure in terms of diet and can give a read out of the metabolic phenotype. Harnessing these complimentary aspects of metabolomics will be key.

To date, the majority of studies in nutritional epidemiology have focused on associations between biomarkers and dietary-intake data, the ultimate challenge is now to move beyond this. Biomarkers, and in particular, endogenous biomarkers, have much more to offer (see Figure 1). To a certain extent, the full potential of metabolomics has been hindered by a focus on associating metabolomics data with dietary data for identification of food-intake biomarkers. We need to embrace linking metabolites to their physiological roles and endogenous metabolic pathways and networks. There is a need to assess the impact of diet on such metabolic pathways and physiological processes, and determine how diet impacts changes in levels of metabolites and consequently health and disease outcomes in prospective cohorts. In the era of precision nutrition, it is also critical to identify and examine biomarkers that reflect the effects of gene–diet or microbiota–diet interactions to help identify subpopulations that may benefit from targeted dietary advice.

A recent study profiled over 1000 metabolites in approximately 11,000 individuals to examine metabolic pathways associated with and across 27 incident noncommunicable diseases (NCDs) [27]. A total of 420 metabolites were shared between at least two NCDs, and metabolic pathway analysis revealed a number of key pathways common across the diseases. A web server was developed, incorporating the results to enable future analysis at the metabolite and pathway level.

With the emergence of rich datasets incorporating multiomics data, the integration of metabolomic data with other omic data is an important approach to obtain functional information. Integration of the blood metabolome with deep immunophenotyping in 500 individuals identified metabolite features associated with eight categories of host factors, including baseline immune parameters and immune cytokine response [28]. Using genome-wide association analysis, a comprehensive landscape of genetic regulation on metabolism was identified. A number of metabolite quantitative trait loci (mQTLs) were identified, and further Mendelian Randomisation analysis revealed that one locus associated with arachidonic acid was causally associated with Crohn’s disease. This study is a pertinent example of how integration of omic datasets provides new insights into the interplay between genes, metabolites and disease risk.

In a UK study of 10,806 participants a total of 66 metabolites were significantly associated with the MDS [22]. These metabolites were combined into a metabolite score which was estimated to explain 37.2% of the inverse association of the Mediterranean diet score with Homeostatic Model of Insulin Resistance (HOMA-IR). The authors suggest that the metabolites including acylcarnitines, sphingolipids and phospholipids act as mediators, and propose their involvement in pathways linking diet to disease risk. This study highlights the potential use of metabolites and is an elegant example of the information we can harness from them.

Using the baseline data for the PREDIMED Randomised Controlled Trial (RCT), a metabolite signature comprising 67 metabolites was associated with the Mediterranean diet adherence score [29]. This signature was replicated in several US cohorts. In prospective analyses, this metabolite signature was predictive of future cardiovascular disease (CVD) risk in both Spanish and US populations. Mendelian Randomization analysis indicated a potential causal relationship between the metabolite signature and CVD risk. Further in-depth study of this metabolic signature and the underlying metabolic pathways will be important for enhancing our understanding of biological mechanisms through which diet impacts CVD.

In a recent analysis that was conducted among more than 9000 free-living individuals participating in five cohort studies, investigators demonstrated that the circulating levels of indolepropionate, a tryptophan metabolite produced by gut bacteria, were robustly associated with a lower type 2 diabetes risk [30]. A further genome-wide association analysis demonstrated that variants in the *LCT* gene that encodes lactase predicted levels of indolepropionate. Interestingly, milk intake significantly interacted with the *LCT* genotype on determining the levels of indolepropionate, and the gut *Bifidobacterium* that was associated with circulating indolepropionate levels in the same study may also potentially mediate such an interaction. In another study conducted in the Health Professionals Follow-Up Study, researchers interrogated human gut microbiomes and identified several species that significantly predicted circulating levels of trimethylamine N-oxide (TMAO) [31]. The species were then used to define a “producer” phenotype, and red meat consumption was associated with TMAO levels only among individuals with the producer phenotype. These studies highlight the role of bioactive metabolites in helping us to deepen our understanding of the gene–diet and microbiome–diet interactions on human health.

Collectively, these studies illustrate the potential of metabolomics to inform on altered metabolic pathways and move towards a systems view of the relationship between food and health. Ultimately, this will help pave the way towards development of Precision Nutrition.

## 4. Future Perspectives for Metabolomics in Nutrition Epidemiology

Metabolomics is one of the most complex omics tools; the added complexity stems from the fact that there is not one set of metabolites making up the metabolome. Indeed, the metabolome is highly dynamic and diverse with metabolites originating external to the host and metabolites that are synthesised endogenously. This added complexity means that the metabolome can be informative both regarding external exposures including dietary intake and on the metabolic phenotype. Acknowledging the dual aspect of the metabolome is key to optimising the potential of metabolomics in nutrition epidemiology.

### 4.1. Improving Self-Reported Dietary Data

The emerging data in terms of biomarkers of food intake is very encouraging. A number of putative biomarkers exist for a range of foods. Validation of these biomarkers will be key to their successful use in nutritional epidemiology. The validation steps require rigorous assessment of the performance of the biomarkers including their stability, reproducibility, time–response and dose–response and are often overlooked in many studies. Giving importance to validation of biomarkers will be key as the field moves forward. Combining self-reported data with metabolomics data is an attractive prospective for addressing measurement error in large-scale studies. Further work is needed to develop statistical tools that will enable the successful modelling of multiple potentially correlated biomarkers with continuous food-intake data. Such models could be used to help infer food-intake data from biomarker data. Furthermore, many biomarkers represent intake over short or medium term. Innovative ways of sampling biospecimens multiple times over a fixed time period to obtain a more comprehensive representation of food intake needs to be developed.

### 4.2. International Efforts for Assignment of Metabolites and Data Sharing

Currently, one of the key bottlenecks in the development of metabolomics for nutrition research is the assignment of new biomarkers. Many of the biomarkers of food intake are exogenous compounds that are not present in spectral databases to aid their assignment. Novel methods are emerging, such as molecular networking, and have the potential to aid metabolite identification. Large-scale international efforts are needed with a focus on food related biomarkers that facilitate data sharing, standard sharing and identification knowhow. Significant advancement in the field of biomarkers for food intake was made in recent years through the collaborative efforts of the FoodBall consortium: a range of systematic reviews were published, as were guidelines on validation of biomarkers [15]. Additionally, international efforts are needed to standardise measurement and reporting procedures to enable reproducibility of findings across cohorts. Furthermore, efforts within the metabolomics community for data sharing need to be embraced in the nutrition epidemiology field. Without a major advancement in this area, it is unlikely that the true potential of metabolomics in terms of biomarkers of food intake will be reached.

### 4.3. Mechanistic Insights

As highlighted above, to optimise the information obtained from metabolomic data we need to progress from using them solely as predictive markers to employ them to inform about altered metabolic pathways and relationships to functional genes and proteins. Using metabolomics in conjunction with orthogonal technologies such as genomics, transcriptomics and proteomics has the potential to model the human response at a systems level. Furthermore, incorporation of microbiome data will be critical to obtain a full systems view. Development of such mechanistic insights will be valuable and informative for the development of precision nutrition.

### 4.4. Translation into Precision Nutrition

Dietary guidelines are often based on population average estimates, which may not be optimal for specific individuals. Precision nutrition is an emerging field that aims to use individualized information, such as data from the genome, microbiome and metabolome, to prescribe personalized diets and lifestyles for chronic disease prevention and management [32]. Metabolomics is an important precision nutrition tool and can help to identify intervention targets and prescribe more personalized nutritional intervention strategies. Using type 2 diabetes as an example, previous studies have found strong and independent positive associations between plasma concentrations of tyrosine, phenylalanine and branched chain amino acids (leucine, isoleucine, and valine) with risk of type 2 diabetes but inverse associations with glycine and glutamine [33]. These findings offer the potential to use dietary modifications to target these metabolites to prevent or treat diabetes. These metabolites can be used to characterize high-risk individuals for interventions and to identify individuals who are responsive to certain intervention strategies. While precision nutrition will likely play an important role in disease prevention and management, the field is still at an early stage, and more studies are needed before its widespread use in clinical and public health settings.

## 5. Concluding Remarks

Metabolomics has great potential in nutritional epidemiology. Harnessing this potential will help address some of the shortcomings of the field. Specifically, the use of food-intake biomarkers can help address measurement error in self-reported dietary intake data and provide a complementary tool to traditional dietary assessment methods. Analysis of endogenous metabolites and metabolic pathways will help move away from a black-box approach to one that delivers information on underlying mechanisms. Integration of metabolomics with orthogonal omic approaches will yield a further understanding of mechanisms at a systems level. For the full realisation of the potential of metabolomics, several key methodological and technological challenges such as food biomarker validations, new metabolite identifications, and integration with other omics have to be addressed. Acknowledging these challenges and building a research roadmap encompassing them will be important in the coming years.
